# High school diploma is associated with longer postpartum leukocyte telomere length in a cohort of primarily Latina women

**DOI:** 10.1186/s40748-024-00193-5

**Published:** 2024-12-03

**Authors:** Dhanya Sumesh, Jue Lin, Janet M Wojcicki

**Affiliations:** 1 Department of Biophysics and Biochemistry, University of California, San Francisco, USA; 2 Division of Pediatric Gastroenterology, Hepatology and Nutrition, Department of Pediatrics, University of California, San Francisco, CA USA; 3 Department of Epidemiology and Biostatistics, University of California, 550 16th Street 4th Floor, San Francisco, CA 94134-0136 USA

## Abstract

**Objective:**

This study investigates correlates of maternal leukocyte telomere length (LTL) in the immediate postpartum period using a cross-sectional study design from an existing prospective longitudinal birth cohort of primarily Latina women. The study focuses on the role of maternal health and dietary habits in pregnancy and maternal education level and LTL at delivery.

**Study Design:**

Latina mothers were recruited during the immediate postpartum period prior to 24 h at two San Francisco hospitals and dried blood spots were collected for LTL analysis via quantitative polymerase chain reaction (qPCR). We used multivariable linear regression models to determine independent predictors of maternal LTL during the postpartum period.

**Results:**

In multivariable regression models, increasing maternal age was associated with shorter LTL during the immediate postpartum period (Coeff − 0.015; *p* < 0.01) whereas having a high school diploma was associated with longer LTL versus not having graduated from high school (Coeff 0.12; *p* < 0.01).

**Conclusion:**

Maternal education level as a potential marker of exposure to life stressors and socioeconomic status was associated with maternal LTL after adjusting for age and other potential confounders in women of reproductive age.

## Introduction

Telomeres, the repetitive DNA sequences at the ends of chromosomes, play a critical role in maintaining chromosomal stability and cellular function [1]. Telomeres become shorter with each cell division and as such they are a biological marker for aging as telomeres get shorter as humans age [2]. Telomeres are also particularly sensitive to inflammation and reactive oxygen species damage, and as such get shorter in the context of chronic disease and exposure to stress [3].

Pregnancy is a pivotal phase in a woman’s life, characterized by complex physiological changes and potential stressors that can influence maternal health and offspring outcomes [4]. Previous studies have explored the potential impact of stress and psychosocial factors on telomere dynamics, suggesting that chronic stress during pregnancy may lead to accelerated fetal and maternal telomere shortening [5]. Moreover, lifestyle factors, including poor nutrition and socioeconomic disparities, and pregnancy-specific morbidities, such as gestational diabetes and preeclampsia, may result in shortened maternal and infant telomeres at delivery and/or throughout the lifecourse [6, 7]. However, the existing literature primarily focuses on predominantly Caucasian or African-American populations with few studies in Latinx populations [8].

Latina women face unique health challenges and disparities including a higher rate of obesity and metabolic disease such as diabetes mellitus compared with other population groups including non-Hispanic whites in the United States [9]. Elucidating the associations between maternal leukocyte telomere length (LTL) and pregnancy outcomes in this group may provide critical knowledge to design targeted interventions and enhance prenatal care strategies. This study investigates the correlates of maternal LTL collected during the immediate postpartum period prior to 24 h, focusing on Latina women with a particular focus on diet and weight gain in pregnancy and sociodemographics. Understanding the correlates of maternal LTL could shed light on the interplay between genetic and environmental factors and thus provide valuable insights into the biological processes and potential health implications during this critical period.

## Methods

We collected data on maternal demographics, health history and reproductive health for 194 participants from two San Francisco hospitals (University of California San Francisco (UCSF) and Zuckerberg San Francisco General Hospital (ZSFG)) as previously described for this longitudinal, primarily Latinx birth cohort [10] In short, all women were recruited and interviewed during the postpartum period and information was extracted from the medical record to confirm all medical diagnoses. Less than 10% of those recruited for the study refused participation. Pre-pregnancy body mass index (BMI (kg/m2)) and weight gain in pregnancy were collected via self-report. We collected pre-pregnancy weight, height and weight at delivery to calculate BMI (weight/height^2) and weight gain in pregnancy (current weight at delivery-pre-pregnancy weight). For participants that were uncertain about weight or height, we extracted data from the medical record. Participants were classified into BMI groups (underweight/normal, overweight and obese) based on the scale outlined by the Centers for Disease Control and Prevention (CDC) [11]. Further, the participants’ pregnancy weight gains were grouped as within, under or over based on CDC pregnancy weight gain groups and American College of Obstetrics and Gynecology (ACOG) guidelines for various BMI groups [12]. Maternal age was categorized into five age groups (18–25, 26–30, 31–35, 36–40, and > 40 years). Dietary intake during pregnancy focused on sugar-sweetened beverage (SSB) consumption including the consumption of sweetened teas, sodas and fruit juices. We also collected self-reported intake of 100% fruit juice consumption. SSB and 100% fruit juice consumption were classified into four groups based on total consumption (in cups per week): none, > 0-<=3 cups, > 3-<=7 cups and > 7 cups for SSB and none, > 0-<=3 cups, > 3-<-5 cups and > 5 cups for 100% fruit juice. Additionally, other variables including parity, marital status (married or living with partner versus single), language use (Spanish versus English), education level (high school diploma versus none) and overall education (high school/GED or less, undergraduate, graduate/postgraduate and other (trade school)) and diabetes mellitus and hypertension in pregnancy were analyzed as categorical variables.

### Leukocyte telomere length analysis

DNA was extracted from the dried blood spots (DBS) on Whatmann 903 cards using the QIAamp DNA Investigator kit (QIAGEN cat# 56504) and eluted in 50 ul ATE buffer. The DBS were collected in three batches: from August 2018 to Nov 2020, from December 2021 to February 2022, and from June 2022 to November 2022. DBS were stored at -80ºC and extracted in December 2020, March 2022, and December 2022 for the respective three batches. LTL assays were performed within a week of DNA extraction. Twenty-four DBS samples from the first batch were re-extracted during the second batch, and 14 samples from the second batch were re-extracted during the third batch to adjust for batch differences. The details of LTL method analysis have been described previously and are a modification of Cawthon’s initial approach [13, 14]. The PCR efficiencies of the T and S reactions are 88.4 ± 4.1% and 91.4 ± 3.9%, respectively. The average inter-assay coefficient of variation (CV) for the samples in this study was 2.0 ± 1.5%. Intra-class correlation (ICC) of repeat extractions of 38 dried blood spot samples from this study was 0.883 (95% CI: 0.766–0.942).

### Statistical analysis

We assessed the normality of our outcome, maternal LTL at delivery finding that the outcome deviated slightly from normality using q-norm and p-norm plots. Transforming the outcome by taking the log of the outcome, we did not see substantial differences in our linear regression comparing the untransformed versus the transformed ones. As such, we used linear regression to examine relationships between maternal LTL and continuous variables such as maternal age (years), age of menarche (years), pre-pregnancy BMI (kg/m2), weight gain in pregnancy (lbs), SSB consumption during pregnancy (cups/week), 100% fruit juice consumption during pregnancy (cups/week), and parity. We also used linear regression to assess the correlation of LTL with categorical variables including the following groups: age BMI, and weight gain and for dichotomous maternal health variables, including depression and anxiety in pregnancy, marital status, gestational hypertension, gestational diabetes, and maternal exposure to smoking.

A multivariable linear regression analysis was performed to assess the independent effects of maternal characteristics on LTL while controlling for potential confounders. Variables that were significant with a *p* < 0.10 and associated with biological plausibility for association with LTL in bivariate analysis (by linear regression) were included in multivariable analysis. The following covariates were included in the multivariable linear regression model: maternal age (years), 100% fruit juice consumption (cups/week), depression and/or anxiety in pregnancy (yes/no), parity (continuous), high school diploma (yes/no), age of menarche (years), and Latina ethnicity (yes/no). All statistical analyses were performed using SAS (Statistical Analysis System), and the results were reported with coefficients and corresponding *p*-values.

## Results

We measured leukocyte telomere length (LTL) in 194 mothers with a mean of 1.44 T/S ratio (SD = 0.28) (Table 1). The age range for mothers was 18 to 41 years, with a mean of 32.05 years (SD = 6.07). Approximately 73% of mothers reported having a high school diploma and 70.1% cited Central America or Mexican ethnicity (Table 1). Overall, 77.3% of the mothers reported some type of Hispanic ethnicity. Eleven point four percent reported depressive symptoms or anxiety in pregnancy and 17.5% had a diagnosis of gestational diabetes mellitus in pregnancy. Thirty point eight percent of the cohort had a pre-pregnancy body mass index (BMI) that was overweight, 26.9% were obese and 42.3% were within the normal range. Weight gain in pregnancy was 20.2% below the ACOG recommendations, 40.4% was within range and 39.3% was above the recommendations. Maternal age showed a significant negative correlation with LTL (Coeff = -0.01, *p* < 0.01) Not possessing a high school diploma was associated with shorter LTL although the results did not meet statistical significance (*p* = 0.06; Table 1).

**Table 1:** Bivariate analysis of LTL related to maternal and infant characteristics (*N* = 194)

*Maternal Characteristics*	*n*	*% of Total*	*Mean ± SD*	*ß (Coeff) 95% CI*	*p-value*
**Leukocyte Telomere Length, T/S ratio**	194		1.44 ± 0.28		
**Maternal Demographics**					
***Maternal Age (cont var), years***			32.05 ± 6.07	**-**0.01 (-0.02-(-)0.004)	**< 0.01**
***Age group***	194				
18–25 yrs	40	20.6	1.57 ± 0.28	1.0	
26–30 yrs	30	15.5	1.47 ± 0.31	-0.10 (-0.23–0 03)	0.13
31–35 yrs	58	29.9	1.40 ± 0.26	-0.17 (-0.28 - -0.05)	**< 0.01**
36–40 yrs	56	28.9	1.37 ± 0.28	-0.19 (-0.38 - -0.08)	**< 0.01**
>40 yrs	10	5.2	1.43 ± 0.27	-0.14 (-0.33–0.05)	0.16
***Marital Status***					
Married/Living with Partner	175	90.2	1.44 ± 0.28	1.0	0.67
Single	19	9.8	1.41±-0.34	-0.03 (-0.17–0.11)	
***Education level***	*192*				
High school/GE or less	61	31.8	1.43 ± 0.31	1.0	
Undergraduate	56	29.2	1.49 ± 0.31	0.06 (-0.05–0.16)	0.28
Graduate/Postgraduate	43	22.4	1.42 ± 0.27	0.05 (-0.12–0.10)	0.85
Other (Trade or Vocational School)	32	16.7	1.37 ± 0.20	-0.06 (-0.19-0.06)	0.31
***High school Diploma***	*194*				
Yes	141	72.7	1.46 ± 0.29	1.0	0.06
No	53	27.3	1.37 ± 0.25	-0.09 (-0.18-0.004)	
***Ethnicity-Country of Origin***	*194*				
Mexican	56	28.9	1.47 ± 0.29	1.0	
Central American	80	41.2	1.40 ± 0.27	-0.07 (-0.17-0.03)	0.15
Other Hispanic	17	8.76	1.49 ± 0.34	0.02 (-0.14-0.17)	0.84
Other Non-Hispanic	41	21.2	1.43 ± 0.29	-0.04 (-0.16-0.07)	0.49
***Latina Ethnicity***	*194*				
Latinas	150	77.3	1.44 ± 0.29	1.0	0.99
Non-Latinas	44	22.7	1.44 ± 0.29	-0.00004 (-0.097-0.096)	
**Language Use**	193				
*English only or Spanish and English*	98	50.78	1.46 ± 0.30	1.0	0.21
*Spanish only*	95	49.22	1.41 ± 0.27	-0.05 (-0.13-0.03)	
**Maternal Health**					
***Depression and/or Anxiety in Pregnancy***	*193*				
No	171	88.6	1.43 ± 0.28	1.0	0.20
Yes	22	11.40	1.51 ± 0.30	0.08 (-0.04-0.21)	
***Hypertension in Pregnancy***	*194*				
No	159	82.0	1.44 ± 0.20	1.0	0.63
Yes	35	18.0	1.42 ± 0.22	-0.03 (-0.13-0.08)	
***Diabetes in Pregnanc*** **y**	*194*				
No	160	82.5	1.44 ± 0.28	1.0	0.77
Yes	34	17.5	1.42 ± 0.31	-0.02 (-0.12-0.09)	
***Menarche Age***,*** years***			12.74 ± 1.63		0.94
***Age of Menarche***	*192*			-0.0009 (-0.02-0.02)	0.94
9–11 years	40	20.8	1.48 ± 0.35	1.0	
12–14 years	125	65.1	1.42 ± 0.26	-0.06 (-0.17-0.04)	0.22
15–17 years	27	14.1	1.48 ± 0.20	-0.01 (-0.15-0.13)	0.93
***Exposure to Secondhand Smoke***	194				0.66
No	181	93.3	1.40 ± 0.10	1.0	
Yes	13	6.7	1.44 ± 0.29	-0.04 (-0.20-0.13)	
**Reproductive History**					
***Pre-pregnancy BMI***,*** kg/m***^***2***^			28.08 ± 7.09		0.78
***Pre-pregnancy BMI***,*** groups***	182				0.65
Normal (BMI > = 18.5–> 25 kg/m2)	77	42.3	1.45 ± 0.27	1.0	
Overweight (BMI 25–30 kg/m2)	49	30.8	1.41 ± 0.31	-0.05 (-0.15-0.06)	0.38
Obese (BMI > 30 kg/m2)	56	26.9	1.42 ± 0.29	-0.03 (-0.13-0.07)	0.55
***Pregnancy weight gain (pounds)***	183	27.9 ± 17.5		-0.0004 (-0.003-0.002)	0.72
***Pregnancy Weight Gain Groups***	183				
In Range for ACOG/IOM	73	40.4	1.42 ± 0.27	1.0	
Above range for ACOG/IOM	72	39.3	1.45 ± 0.29	0.03 (-0.07-0.12)	0.58
Below Range for ACOG/IOM	37	20.2	1.44 ± 0.33	0.02 (-0.09-0.14)	0.72
***Sugar-Sweetened Beverage Intake***,*** cups/week***	*194*		2.36 ± 3.58	-0.004 (-0.02-0.007)	0.48
None	78	40.2	1.43 ± 0.28	1.0	
*>0 cups and < = 3 cups*	73	37.6	1.47 ± 0.30	0.03 (-0.06-0.13)	0.47
*>3 cups and < = 7 cups*	27	13.9	1.40 ± 0.24	-0.03 (-0.16-0.10)	0.63
>7 cups	16	8.3	1.39 ± 0.31	-0.04 (-0.20-0.11)	0.58
***100% Fruit Juice Intake***,*** cups/week***	194		2.16 ± 3.72	0.002 (-0.009-0.013)	0.69
None	84	43.3	1.44 ± 0.29	1.0	
*>0 cups and < = 3 cups*	69	35.6	1.42 ± 0.27	-0.02 (-0.11-0.07)	0.69
*>3 cups and < = 5 cups*	20	10.3	1.39 ± 0.35	-0.05 (-0.19-0.09)	0.49
>5 cups	21	10.8	1.53 ± 0.28	0.09 (-0.04-0.23)	0.18
***Parity***			2.06 ± 0.99	-0.03 (-0.07-0.009)	0.13
**Infant Characteristics**					
***Child’s Sex***	194				
Male	115	59.3	1.42 ± 0.26	1.0	
Female	79	40.7	1.46 ± 0.32	0.04 (-0.13-0.04)	0.29

**Table 2:** Multivariable analysis of predictors of Postpartum maternal leukocyte telomere length (*n* = 190)

Maternal Characteristics	Coefficients, 95% CI	*P*-value
***Maternal age***,*** years***	-0.015 (-0.02-(-) 0.007)	**< 0.01**
***100% Fruit Juice Consumption***,*** cups***	0.001 (-0.009-0.012)	0.79
***Depression and/or Anxiety in Pregnancy***		
No	1.00	
Yes	0.049 (-0.08-0.18)	0.45
***Parity***	0.005 (-0.039-0.049)	0.82
***High School Diploma***		
No	1.00	
Yes	0.12 (0.02–0.22)	**0.02**
***Age of Menarche***,*** years***	-0.002 (-0.03-0.02)	0.87
***Maternal Ethnicity***		
*Non-Latina*	1.00	
Latina	0.05 (-0.06-0.16)	0.36

### Multivariable regression

In a multivariable regression model that included maternal age (years), 100% fruit juice consumption in pregnancy (cups/week), depression and/or anxiety in pregnancy (yes/no), parity, high school diploma status (yes/no), age of menarche (years) and ethnicity (Latina versus non-Latina), increasing maternal age was an independent predictor of shorter LTL (β =-0.015, p = < 0.01; Table 2) and having a high school diploma was associated with longer LTL (β = 0.12, *p* = 0.02;  Table 2).

When the analysis was restricted to only Latina women, high school diploma was still associated with longer LTL but the effect size was larger and *p* value was more significant (β = 0.13, *p* = 0.01; results not shown).

## Discussion

Our results with this cohort of Latina postpartum women confirm the finding from other studies indicating that advanced maternal age is linked to shorter LTL [2, 15]. Every time a cell divides, a portion of the telomeric DNA fails to replicate and as such older organisms will have shorter telomeres [16]. Additionally, the cumulative exposure to oxidative stress, inflammation and psychosocial stress can do cellular damage over time resulting in telomere attrition [17].

We also found a that mothers with a high school diploma had longer LTL than those without a high school diploma; the strength of the association was greater among Latina mothers but present for all mothers in our cohort. We have previously found an association between lower maternal education and shorter LTL at birth [18] among neonates but our previous studies did not find an association between maternal LTL and education level. Meanwhile, our findings are in line with other studies that have demonstrated an association between lower socioeconomic status (SES) and education levels and shorter LTL [19, 20]. Individuals with lower SES and/or education levels experience more intense exposure to risk factors which could accelerate LTL attrition including obesity, food insecurity, exposure to secondhand smoke, and lack of exercise [21].

One recent study addressed some of the potential genetic and early life confounders of LTL, SES and education level by studying LTL of siblings with different educational attainment finding that high school and college graduates had longer LTL than those that did not obtain a high school diploma [22] Meanwhile this study was conducted with individuals of European ancestry in contrast to our with primarily Latinas; the interplay between SES and education may differ based on racial/ethnic background as well as immigration status. We did not find any association between education level and LTL when education was characterized as a multi-level variable versus dichotomous (high school diploma versus none). Other studies have similarly found this education cut-point (high school diploma) important to differentiate health outcomes including one of our studies focused on LTL in neonates [18, 23].

### Future directions and limitations

Future research could delve deeper into the mechanisms through which educational attainment influences LTL. Exploring potential mediators, such as stress, inflammation, and components of SES such as housing and food security and exposures to environmental pollutants, may offer a more nuanced understanding of these relationships [24].

Limitations of this study include small sample size and the cross-sectional design that restricts our ability to draw causal inferences [25]. We also did not collect any information on immigration status and length of residence in the United States which could have confounded the relationship between maternal education and LTL [26]. Recent immigrants face significant stressors including discrimination and history of trauma that could impact LTL shortening. Formal education level may serve as a buffer against certain types of immigration stressors [26]. Other predictors of LTL including weight gain in pregnancy and symptoms of depression/anxiety were collected mainly via self-report versus medical chart extraction or assessment using standardized instruments. Further research, ideally longitudinal in nature, could provide more insights into the temporal relationships of how education impacts LTL by assessing possible mediators. Additionally, the study’s focus on the Latina population in the Bay Area reflects the demographics of the Bay Area’s Latina population (mainly Mexican and Central American origin) and may limit the generalizability of findings to other populations including other Latina populations.

## Data Availability

All requests for access to data should be made to to the corresponding author (JMW).

## References

[1] Panelli DM, Bianco K. Cellular aging and telomere dynamics in pregnancy. Curr Opin Obstet Gynecol. 2022;34(2):57–61. 10.1097/GCO.0000000000000765. PMID: 34845136; PMCID: PMC8891073. 10.1097/GCO.0000000000000765 PMC8891073 34845136

[2] Vaiserman A, Krasnienkov D. Telomere length as a marker of Biological Age: state-of-the-Art, Open Issues, and future perspectives. Front Genet. 2021;11:630186. 10.3389/fgene.2020.630186. PMID: 33552142; PMCID: PMC7859450. 33552142 10.3389/fgene.2020.630186 PMC7859450

[3] Barnes RP, Fouquerel E, Opresko PL. The impact of oxidative DNA damage and stress on telomere homeostasis. Mech Ageing Dev. 2019;177:37–45. 10.1016/j.mad.2018.03.013. Epub 2018 Mar 28. PMID: 29604323; PMCID: PMC6162185. 29604323 10.1016/j.mad.2018.03.013 PMC6162185

[4] Valsamakis G, Chrousos G. George Mastorakos. Stress, female reproduction, and pregnancy. 2019;100:48–57. 10.1016/j.psyneuen.2018.09.031 30291988

[5] Moshfeghinia R, Torabi A, Mostafavi S, et al. Maternal psychological stress during pregnancy and newborn telomere length: a systematic review and meta-analysis. BMC Psychiatry. 2023;23:947. 10.1186/s12888-023-05387-3. 38102621 10.1186/s12888-023-05387-3 PMC10724935

[6] Lazarides C, Epel ES, Lin J, Blackburn EH, Voelkle MC, Buss C, Simhan HN, Wadhwa PD, Entringer S. Maternal pro-inflammatory state during pregnancy and newborn leukocyte telomere length: a prospective investigation. Brain Behav Immun. 2019;80:419–26. 10.1016/j.bbi.2019.04.021. Epub 2019 Apr 8. PMID: 30974172; PMCID: PMC7954441. 30974172 10.1016/j.bbi.2019.04.021 PMC7954441

[7] Harville EW, Williams MA, Qiu C, et al. Telomere length, pre-eclampsia, and gestational diabetes. BMC Res Notes. 2010;3:113. 10.1186/1756-0500-3-113. 20416088 10.1186/1756-0500-3-113 PMC2873349

[8] Jones CW, Gambala C, Esteves KC, Wallace M, Schlesinger R, O’Quinn M, et al. Differences in placental telomere length suggest a link between racial disparities in birth outcomes and cellular aging. Am J Obstet Gynecol. 2017;216(3):294.e1–294.e8. 10.1016/j.ajog.2016.11.1027. Epub 2016 Nov 16. PMID: 27865975; PMCID: PMC5334179. 10.1016/j.ajog.2016.11.1027 PMC5334179 27865975

[9] Alemán JO, Almandoz JP, Frias JP, Galindo RJ. Obesity among latinx people in the United States: a review. Obes (Silver Spring). 2023;31(2):329–37. 10.1002/oby.23638. PMID: 36695058; PMCID: PMC9937439. 10.1002/oby.23638 PMC9937439 36695058

[10] Prasad A, Lin J, Jelliffe-Pawlowski L, Coleman-Phox K, Rand L, Wojcicki JM. Sub-optimal maternal gestational gain is associated with shorter leukocyte telomere length at birth in a predominantly latinx cohort of newborns. Maternal Health Neonatal Perinat. 2023;9(1):14. 10.1186/s40748-023-00167-z. PMID: 37919818; PMCID: PMC10623801. [Ref]. 10.1186/s40748-023-00167-z PMC10623801 37919818

[11] Centers for Disease Control and Prevention, Adult BMI, Categories. https://www.cdc.gov/bmi/adult-calculator/bmi-categories.html, accessed September 17, 2024.

[12] American College of Obstetricians and Gynecologists. ACOG Committee opinion no. 548: weight gain during pregnancy. Obstet Gynecol. 2013;121(1):210-2. 10.1097/01.aog.0000425668.87506.4c. PMID: 23262962. 10.1097/01.aog.0000425668.87506.4c 23262962

[13] Cawthon RM. Telomere measurement by quantitative PCR. Nucleic Acids Res. 2002;30(10):e47. 10.1093/nar/30.10.e47. PMID: 12000852; PMCID: PMC115301. 12000852 10.1093/nar/30.10.e47 PMC115301

[14] Lin J, Epel E, Cheon J, Kroenke C, Sinclair E, Bigos M, Wolkowitz O, Mellon S, Blackburn E. Analyses and comparisons of telomerase activity and telomere length in human T and B cells: insights for epidemiology of telomere maintenance. J Immunol Methods. 2010;352(1–2):71–80. Epub 2009 Oct 21. PMID: 19837074; PMCID: PMC3280689. 19837074 10.1016/j.jim.2009.09.012 PMC3280689

[15] Shammas MA. Telomeres, lifestyle, cancer, and aging. Curr Opin Clin Nutr Metab Care. 2011;14(1):28–34. 10.1097/MCO.0b013e32834121b1. PMID: 21102320; PMCID: PMC3370421. 21102320 10.1097/MCO.0b013e32834121b1 PMC3370421

[16] Olovnikov AM. A theory of marginotomy. The incomplete copying of template margin in enzymic synthesis of polynucleotides and biological significance of the phenomenon. J Theor Biol. 1973;41(1):181 – 90. 10.1016/0022-5193(73)90198-7. PMID: 4754905. 10.1016/0022-5193(73)90198-7 4754905

[17] Epel ES, Blackburn EH, Lin J, Dhabhar FS, Adler NE, Morrow JD, Cawthon RM. Accelerated telomere shortening in response to life stress. Proc Natl Acad Sci U S A. 2004;101(49):17312–5. 10.1073/pnas.0407162101. Epub 2004 Dec 1. PMID: 15574496; PMCID: PMC534658. 15574496 10.1073/pnas.0407162101 PMC534658

[18] Wojcicki JM, Olveda R, Heyman MB, Elwan D, Lin J, Blackburn E, Epel E. Cord blood telomere length in latino infants: relation with maternal education and infant sex. J Perinatol. 2016;36(3):235–41. 10.1038/jp.2015.178. Epub 2015 Dec 3. PMID: 26633142. 26633142 10.1038/jp.2015.178

[19] Cherkas LF, Aviv A, Valdes AM, Hunkin JL, Gardner JP, Surdulescu GL, Kimura M, Spector TD. The effects of social status on biological aging as measured by white-blood-cell telomere length. Aging Cell. 2006;5(5):361–5. 16856882 10.1111/j.1474-9726.2006.00222.x

[20] Steptoe A, Hamer M, Butcher L, Lin J, Brydon L, Kivimäki M, Marmot M, Blackburn E, Erusalimsky JD. Educational attainment but not measures of current socioeconomic circumstances are associated with leukocyte telomere length in healthy older men and women. Brain Behav Immun. 2011;25(7):1292–8. Epub 2011 Apr 23. PMID: 21536122. 21536122 10.1016/j.bbi.2011.04.010

[21] Adler N, Pantell MS, O’Donovan A, Blackburn E, Cawthon R, Koster A, Opresko P, Newman A, Harris TB, Epel E. (2013). Educational attainment and late life telomere length in the Health, Aging and Body Composition Study. Brain, Behavior, and Immunity, 27, 15–21. 10.1016/j.bbi.2012.08.014 PMC3543785 22981835

[22] Amin V, Fletcher JM, Sun Z, Lu Q. Higher educational attainment is associated with longer telomeres in midlife: evidence from sibling comparisons in the UK Biobank. SSM Popul Health. 2021;17:101018. 10.1016/j.ssmph.2021.101018. PMID: 35024423; PMCID: PMC8728101. 35024423 10.1016/j.ssmph.2021.101018 PMC8728101

[23] Rhew IC, Catalano RF, Hawkins JD. Mechanisms linking high school graduation to health disparities in young adulthood: a longitudinal analysis of the role of health behaviours, psychosocial stressors, and health insurance. Public Health. 2016;139:61–9. 10.1016/j.puhe.2016.06.010. Epub 2016 Jul 6. PMID: 27395333; PMCID: PMC5061606. 27395333 10.1016/j.puhe.2016.06.010 PMC5061606

[24] Lin J, Epel E. Stress and telomere shortening: insights from cellular mechanisms. Ageing Res Rev. 2022;73:101507. 10.116/j.arr.2021.101507. Epub 2021 Nov 1. PMID: 34736994; PMCID: PMC8920518. 34736994 10.1016/j.arr.2021.101507 PMC8920518

[25] Setia MS. Methodology Series Module 3: cross-sectional studies. Indian J Dermatol. 2016 May-Jun;61(3):261–4. 10.4103/0019-5154.182410. PMID: 27293245; PMCID: PMC4885177. 10.4103/0019-5154.182410 PMC4885177 27293245

[26] Sanchez M, Diez S, Fava NM, Cyrus E, Ravelo G, Rojas P, Li T, Cano MA, De La Rosa M. Immigration stress among recent latino immigrants: the protective role of Social Support and Religious Social Capital. Soc Work Public Health. 2019;34(4):279–92. Epub 2019 Apr 29. PMID: 31033427; PMCID: PMC9872174. 31033427 10.1080/19371918.2019.1606749 PMC9872174

